# Colonic Adaptation Postileal Resection: Bile Acid Absorption in an Ileal Resection Mouse Model

**DOI:** 10.1155/grp/2664210

**Published:** 2025-11-10

**Authors:** Yudai Goto, Kouji Masumoto, Takato Sasaki, Kentaro Ono, Hiroshi Ohno, Masaya Araki, Takashi Matsuzaka, Hitoshi Shimano

**Affiliations:** ^1^Department of Pediatric Surgery, Faculty of Medicine, University of Tsukuba, Tsukuba, Ibaraki, Japan; ^2^Department of Endocrinology and Metabolism, Faculty of Medicine, University of Tsukuba, Tsukuba, Ibaraki, Japan; ^3^Graduate School of Comprehensive Human Sciences, University of Tsukuba, Tsukuba, Ibaraki, Japan; ^4^Division of Complex Biosystem Research, Department of Research and Development, Institute of Natural Medicine, University of Toyama, Toyama, Toyama, Japan

**Keywords:** bile acid absorption, colonic adaptation, functional adaptation, intestinal adaptation, short bowel syndrome

## Abstract

**Background:**

Adaptation of the small intestine and/or colon significantly impacts the prognosis of short bowel syndrome. This study investigated colonic adaptation in a mouse model of ileal resection, with a focus on bile acid absorption.

**Methods:**

The ileal resection mouse model (ileal resection group, 8–10-week-old male C57BL/6J mice) was created by resecting 15 cm of the ileum, corresponding to approximately 50% of the small intestine, while preserving the cecum. The sham group underwent intestinal transection and reanastomosis at a site matched in distance from the ligament of Treitz to that used for the resection group. Postoperatively, between Days 1–7 and 7–14, mice received the elemental diet ELENTAL® (0.5 kcal/mL) and a standard solid diet ad libitum, respectively. The mice were euthanized on Day 14. We assessed postoperative body weight; histopathological characteristics of the colon; bile acid metabolism-related gene expression, including *Asbt* for luminal bile acid uptake, *Fabp6* for cytosolic transport, Ostb for bile acid excretion into the circulation, and Fxr, the primary intracellular bile acid receptor regulating the genes; and fecal and serum bile acid concentrations.

**Results:**

Significantly lower changes in body weight and longer colon length were observed in the ileal resection group than in the sham group; however, no histological differences were observed in colonic mucosal height. Furthermore, a significantly increased *Asbt* expression was detected in the ileal resection group. No significant differences were observed in bile acid concentrations in the feces and serum in both groups.

**Conclusion:**

Our results suggest a colonic adaptation to prevent impairment of bile acid absorption following ileal resection.

## 1. Introduction

Short bowel syndrome (SBS), characterized by a substantial reduction in intestinal length owing to surgical resection, congenital defects, or disease-associated loss, disrupts normal nutrient absorption, leading to severe malnutrition and metabolic complications. The management of SBS presents considerable challenges and necessitates a multidisciplinary approach. Despite advances in surgical techniques and nutritional support, the long-term prognosis and quality of life in patients with SBS remain significantly compromised. Consequently, exploring the mechanisms underlying intestinal adaptations, including colonic adaptations, after extensive bowel loss is critical [1].

Intestinal adaptation is a compensatory physiological process following the loss of mucosal surface area, resulting in the enhancement of both the structure and function in the residual bowel and the restoration of the digestive and absorptive capacities in the intestine [1]. Functional changes in intestinal adaptation are mediated by two mechanisms [2]: an adaptive response associated with an increase in cells relevant to the function [3, 4] and changes in cellular functions and overall structural adaptations [5]. Understanding the process of functional adaptation and identifying the factors that influence this process is crucial for determining prognosis and therapeutic interventions for patients with SBS.

The clinical outcome in patients with SBS depends on several factors, including the length of the remaining intestine, underlying intestinal disease, functional status of other digestive organs, and the anatomical site of resection [2]. Ileocecal resection (ICR), the most common type of resection [1], generally results in more severe conditions than jejunal resection [6], which is attributed to the lower adaptive capabilities of the duodenum and jejunum compared to the ileum in an adaptive response to intestinal resection [7]. Additionally, the disruption of bile acid (BA) metabolism is one of the most severe complications of ileal loss, leading to diarrhea, consequent fluid loss, and malabsorption of fats [8], and may contribute to intestinal failure-associated liver disease [9].

Intestinal resection and anastomosis in mice have been performed by limited research groups, which investigated the fundamental physiological processes underlying adaptation at the cellular level, including regulation of mucosal proliferation, apoptosis, transport, and digestive enzyme expression [1, 10–12]. Rapid advancements in mouse models related to specific genes may have significant opportunities for future research on the mechanisms of SBS. These models are particularly valuable for identifying the contributions of specific molecules that mediate the process of intestinal adaptation and for elucidating the differences in prognosis under various conditions and the significance of therapeutic interventions [13].

One widely used mouse model of small bowel resection involves resection of the proximal portion (jejunum) while preserving the ileum and colon [10, 14]. Another one is the ICR model, which reflects the primary group of human patients with SBS [2, 11]. In distal small intestinal resections, the cecum and at least 1–2 cm of the proximal colon are generally removed; however, reports using an ileal resection (IR) model, in which the ileal valve is preserved in mice, as shown in our study, have not yet been published.

We hypothesized that functional adaptation occurs in the colon of the mouse IR model, preserving the ileocecal valve. To test this hypothesis, we investigated the morphological intestinal changes and crucial functional changes in the expression of genes associated with BA circulation. The following genes were specifically chosen for their roles in the uptake of BAs by ileal enterocytes (*Asbt*), their intracellular shuttling (*Fabp6*), and their extrusion into the circulation (*Ostb*) based on previous literature [15].

## 2. Materials and Methods

### 2.1. Animals and Diets

Eight-week-old male wild-type (WT) C57BL/6J mice were obtained from CLEA Japan (Tokyo, Japan). Mice were housed in a pathogen-free barrier facility under a 12 h light/dark cycle at constant temperature (23°C) and provided with water and a solid standard rodent diet ad libitum (MF, Oriental Yeast, Tokyo, Japan). The mice were divided into two groups, IR and sham, according to the surgical procedure.

This study was approved by the Animal Care Committee of the University of Tsukuba, Japan, and performed in accordance with its guidelines. All animal husbandry and experimental protocols conformed to the University of Tsukuba Regulations for Animal Experiments and were approved by the Animal Experiment Committee of the University of Tsukuba. All experiments were executed according to the ARRIVE guidelines.

### 2.2. Surgical Procedure

**Figure 1:**
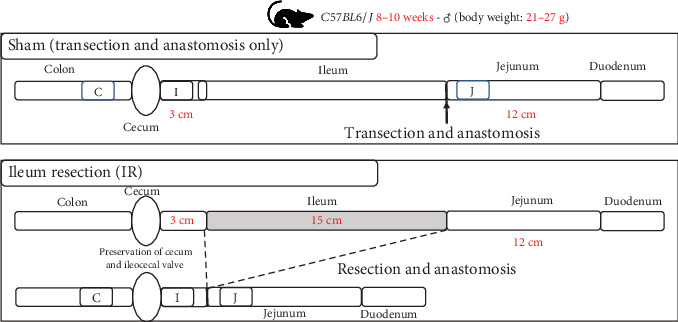
Overview of surgical procedures in the sham and ileal resection (IR) groups. J, jejunum; I, ileum; C, colon.

At 8 weeks of age, the mice were randomly assigned to undergo either IR (*n* = 9) or a sham operation (*n* = 9). At 24 h before the operation, mice were switched from a solid standard rodent diet to a liquid elemental diet (ELENTAL, EA Pharma Corporation, Tokyo, Japan). Preoperatively, mice were weighed and placed on a heating pad. Under isoflurane anesthesia, a midline laparotomy was performed, and the bowel was externalized. In the IR group, the proximal 15 cm was measured 3 cm from the end of the ileum, a window was made in the mesentery, and the mesenteric vessels supplying the segment of the small bowel to be resected were ligated. Then, the 15 cm ischemic ileal bowel was resected, corresponding to approximately 50% of the small intestine. A handsewn Albert–Lembert anastomosis was performed using 9-0 nylon under a microscope. In the sham group, bowel transection was performed at a site 12 cm distal to the ligament of Treitz, and reanastomosis was performed without bowel resection (Figure 1). All surgeries were performed by a single surgeon to minimize variability due to differences in surgical technique.

Buprenorphine was injected into the subcutaneous space to control postoperative pain. Water was provided ad libitum for the first 24 h after surgery; after this period, mice were fed a liquid elemental diet (concentration: 0.5 kcal/mL) ad libitum from the postoperative day (POD) 1 to POD 7. On POD 8, the mice were fed a standard rodent diet ad libitum. Food consumption and body weight were measured daily after surgery. On POD 14, the mice were sacrificed by cervical dislocation while under isoflurane anesthesia. The remnant intestinal length was measured, and the ends of the jejunum (1 cm proximal from the anastomosis), ileum (1 cm proximal from the cecum), and colon (1 cm distal to the cecum) were collected. One centimeter of the intestinal length, adjacent to the anastomosis site, was discarded because of the possible inflammatory changes resulting from the surgical procedure. The intestinal samples were obtained on the antimesenteric side and washed with cold saline.

### 2.3. Histological Analysis

The jejunum, ileum, and colon were formalin-fixed, embedded in paraffin, sectioned at 5 *μ*m thickness, and stained with hematoxylin and eosin (H&E). To assess morphological intestinal adaptation, villous height and crypt depth were measured in each sample, as previously described [16]. All the sections were visualized under a microscope (BZ-X710; Keyence, Osaka, Japan). At least 20 well-oriented villi and crypts from the jejunum or ileum were assessed in each sample. Additionally, in the colon, the thickness of the tunica mucosa coli per sample was also measured. All samples were analyzed using the software provided with the microscope (BZ analyzer, Version 3.6.0.0, Keyence) by a single-blinded researcher to eliminate selection bias while maintaining consistency in the length-counting methods.

### 2.4. RNA Purification and Real-Time PCR

Total RNA was extracted from frozen mouse tissues using Sepasol-RNA I Super G (Nacalai Tesque). Total RNA was reverse transcribed using the PrimeScript RT Master Kit (Takara Bio Inc., Shiga, Japan). Real-time PCR was performed using the Thermal Cycler Dice Real-Time System Single (Takara Bio Inc., Shiga, Japan) and TB Green Premix Ex Taq II (Takara Bio Inc.). mRNA expression was normalized to the *Gapdh* mRNA content and presented as a fold change compared to that in sham mice using the *ΔΔ*CT method. The primer sequences are listed in Table 1. *Asbt* was analyzed for luminal BA uptake, *Fabp6* for cytosolic transport, *Ostb* for BA export into circulation, and *Fxr*, the primary intracellular BA receptor that regulates these genes [15].

### 2.5. Plasma Analysis

Plasma and BA outputs were analyzed as described previously [17]. Plasma total BA (TBA) levels were measured using enzymatic kits (Wako Pure Chemical Industries, Osaka, Japan). At 24 h before sacrifice, the mice were individually housed for fecal collection. The feces were dried, weighed, and crushed into a powder. Fecal BAs were extracted from powdered feces using 90% ethanol, and their concentrations were determined enzymatically using a TBA kit (Wako Pure Chemical Industries, Osaka, Japan).

### 2.6. Statistical Analysis

All data were expressed as means with 95% confidence intervals (CIs). Because of the small sample size and the nonnormal distribution of data, nonparametric Mann–Whitney *U* tests were used for statistical analyses. A simple linear regression analysis was performed to investigate mRNA expression correlation. All statistical analyses were performed using the GraphPad Prism Version 9.5.1 (San Diego, California, United States). *p* value of less than 0.05 was considered statistically significant.

## 3. Results

### 3.1. Postoperative Body Weight and Food Intake

**Figure 2:**
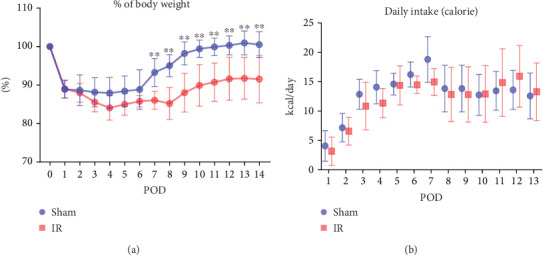
Postoperative body weight and daily caloric intake in the sham and ileal resection (IR) groups (*n* = 9). (a) Postoperative changes in body weight. (b) Daily caloric intake. ⁣^∗∗^*p* < 0.01.

The recovery of postoperative body weight showed an apparent delay in the IR group, compared to that in the sham group (Figure 2a). In the specific change in body weight in the sham group, although the body weight initially decreased postoperatively, it started to recover on POD 7 and returned to preoperative levels by POD 14. In contrast, the IR group showed weight gain from POD 9 onwards; however, on POD 14, the body weight had not fully returned to preoperative levels, with a mean value of 93% of the preoperative weight. No significant differences in food intake were observed between the two groups (Figure 2b).

### 3.2. Morphological Changes in the Jejunum, Ileum, and Colon

**Figure 3:**
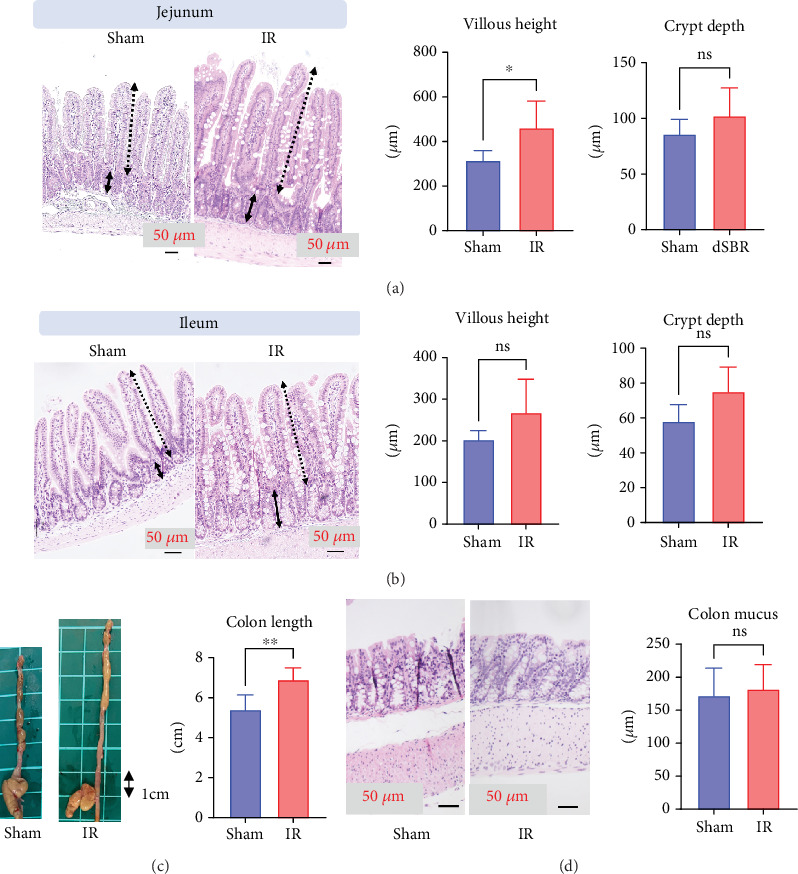
Macroscopic and histopathological findings of the jejunum, ileum, and colon in the sham and ileal resection (IR) groups on Postoperative Day 14 (n = 9). (a) Jejunal mucosa. (b) Ileal mucosa. (c) Gross appearance of the colon length. (d) Colonic mucosal surface. Sections are stained with hematoxylin and eosin (20× magnification). ⁣^∗^*p* < 0.05, ⁣^∗∗^*p* < 0.01.

In the residual jejunum, the villus height was significantly increased in the IR group, compared to that in the sham group (mean IR group: 461 *μ*m vs. sham group: 315 *μ*m, *p* = 0.01); however, there were no significant differences observed in crypt depth between the two groups (mean IR group: 102 *μ*m vs. sham group: 86 *μ*m, *p* = 0.15) (Figure 3a).

In the residual ileum, no significant differences were observed in villus height or crypt depth between the two groups (villus height: IR group mean, 268 *μ*m vs. sham group mean, 203 *μ*m, *p* = 0.05; crypt depth: IR group mean, 75 *μ*m vs. sham group mean, 58 *μ*m, *p* = 0.15) (Figure 3b).

The colon length in the IR group was significantly greater than that in the sham group (IR group mean: 6.9 cm vs. sham group: 5.4 cm, *p* = 0.007) (Figure 3c); however, there were no significant differences in the height of the colonic mucosal surface between the two groups, with the IR and sham groups having a mean of 182 and 172 *μ*m (*p* = 0.77), respectively (Figure 3d).

### 3.3. Differential Expression of BA Metabolism Genes in the Jejunum, Ileum, and Colon

**Figure 4:**
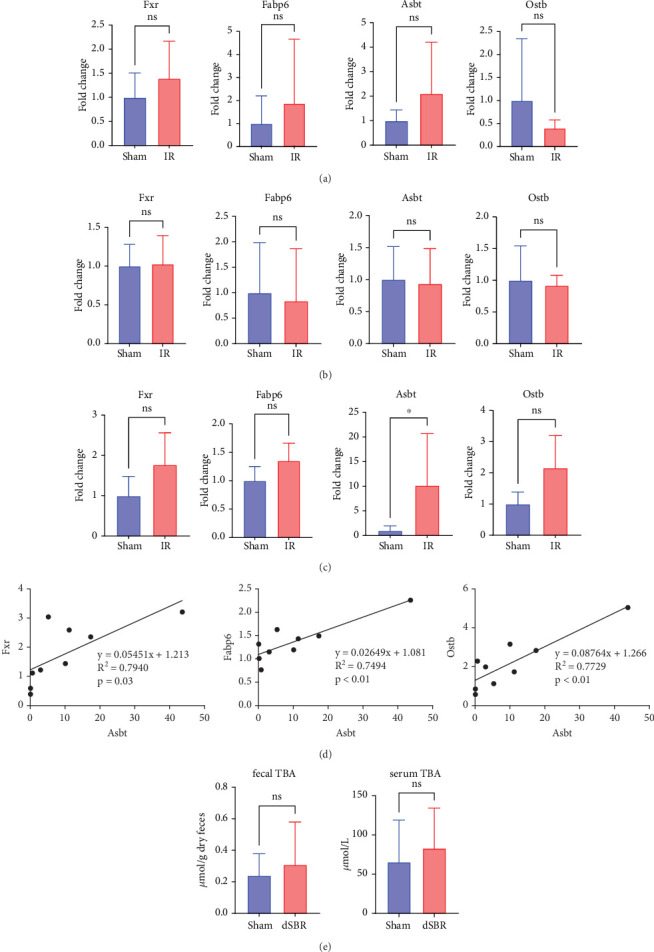
Expression of bile acid (BA) absorption-related genes and total BA (TBA) levels in the sham and ileal resection (IR) groups (*n* = 9). (a) Expression levels of Fxr, Fabp6, Asbt, and Ostb in the residual jejunum. (b) Expression levels of Fxr, Fabp6, Asbt, and Ostb in the residual ileum. (c) Expression levels of Fxr, Fabp6, Asbt, and Ostb in the colon, 1 cm distal to the cecum. (d) Correlation analysis between Asbt expression and other BA absorption-related genes. (e) Fecal and serum TBA concentrations in the sham and IR groups ⁣^∗^*p* < 0.05.

We investigated changes in BA metabolism in the residual jejunum following IR. In the IR group, the expression levels of *Fxr*, *Fabp6*, *Asbt*, and *Ostb* showed no significant differences between the two groups (*p* = 0.58, *p* = 0.67, *p* = 0.86, and *p* = 0.14) (Figure 4a). Additionally, in the residual ileum, there were no differences in these expression levels between the IR and sham groups (*p* = 0.82, *p* = 0.43, *p* = 0.82, and *p* = 0.93) (Figure 4b).

Next, a similar investigation was conducted to determine changes in BA metabolism in the colon, 1 cm distal to the cecum. *Asbt* expression was significantly increased in the IR group compared with that in the sham group (*p* = 0.03; Figure 4c). Although no statistical differences were observed in *Fxr*, *Fabp6*, and *Ostb* expression levels between the IR and sham groups (*p* = 0.08, *p* = 0.07, and *p* = 0.05, respectively; Figure 4c), the gene expression levels were correlated with the *Asbt* expression levels (Figure 4d).

### 3.4. BA Concentration in the Plasma and Feces

We found no significant differences in fecal BA concentrations between the two groups, with mean values of 0.24 *μ*mol/g dry feces in the sham group and 0.31 *μ*mol/g dry feces in the IR group (*p* = 0.96) (Figure 4e). Moreover, there were no significant differences in serum BA concentrations between the two groups, with mean values of 65 *μ*mol/L in the sham group and 83 *μ*mol/L in the IR group (*p* = 0.39) (Figure 4e).

## 4. Discussion

In this study, a mouse model of IR with preservation of the ileocecum was used to evaluate postoperative functional adaptation in the colon by analyzing gross and histological findings and the expression of genes related to BA absorption. The IR group had significantly longer colons and an increased expression of genes related to BA absorption. Within the residual jejunum and ileum in the IR group, there was no obvious increase in the expression of BA absorption-related genes compared to the sham group, although morphological changes in the jejunum were consistent with postoperative intestinal adaptation. These findings suggest that the colon may play a pivotal role in intestinal adaptation after IR.

In this study, an increase in the colon length was demonstrated in a mouse IR model in which the ileum was resected while preserving the cecum. This finding is consistent with previous reports on morphological changes occurring in the colons of rat models after IR or ICR. Jiang et al. reported that, in rats that underwent a 90%–95% small intestine resection, there were significant increases in the length, diameter, and height of mucosal folds, as well as in colonic mucosal thickness, compared to those in controls [18]. Moreover, Soler et al. also found that rats with 70% intestinal resection, especially those with preserved ileocecal valves, showed morphological adaptation to the colonic mucosa [19]. The colonic changes, as shown in those studies and our results, indicate that preserving intestinal structures, such as the ileocecal valve, might play a crucial role in the adaptive response of the colon following massive small bowel resection. In contrast, studies in mice by Matsumoto et al. showed that massive small intestinal resection, including ICR, led to an increase in the diameter of the proximal colon compared to nonsurgical cases, with no significant difference from the sham group and without a change in the length of the colon among any of the groups [2]. Although no studies have focused on the morphological changes in the colon after IR and ICR in mice, considering that our model was a cecum-sparing IR one, this discrepancy might indicate differences in the extent and location of the massive small intestinal resection.

Several studies have shown that both morphological and functional changes occur in the colon of mouse models of ICR. Previous studies in which 12 cm of the ileum, cecum, and 1–2 cm of the proximal colon were resected in mice showed increased expression of BA-related genes, *Fabp6* and *Asbt*, in colonocytes [2, 15]. To explain the upregulation of BA absorption-related genes in the colon following ICR, several hypotheses have been proposed. Healey et al. proposed that the increased absorption of BAs serves as a protective mechanism against the adverse effects of excess BAs, such as heightened water and electrolyte secretion, severe nutrient loss, and diarrhea [20]. Dekaney et al. posited that an elevated BA load in the colonic lumen might be the primary stimulus for *Fxr*-dependent transcription, thereby enhancing the expression of genes such as *Fabp6* in colonocytes following ICR [15]. Matsumoto et al. stated that mRNA levels of BA metabolism-related factors, specifically *Fabp6*, *Asbt*, and *Ostb*, showed an increase in the colon in the ICR model in mice and concluded that the epithelium of the remnant intestine, particularly that of the proximal colon, responds to the loss of the distal small intestine by altering the expression levels of genes involved in BA absorption. In addition, certain BA-related genes, including *Fabp6* and *Ostb*, are also induced in the distal colon, suggesting that the direct exposure of the remaining colon to elevated concentrations of luminal BAs is likely the predominant mechanism driving the upregulation of genes essential for BA absorption [2].

The hypothesis that the upregulation of BA-related genes in the colon is related to intestinalization of the colon is controversial. Tecos et al. reported that the changes to villous-like structures in the colon after ICR in mice were correlated with functional adaptation, based on findings of increased expression of sucrase isomaltase, an enzyme specific to the small intestinal villi [21]. However, Matsumoto et al. reported that the adaptive response to vitamin B12 absorption in the colon was absent following ICR [2], suggesting that the upregulation of genes related to BA absorption may not be due to “intestinalization” of the colon after IR. Therefore, to clarify whether functional adaptation of the colon or intestinalization occurs after IR, a detailed examination of each nutrient absorption in the remaining colon may be necessary.

Recent advances in glucagon-like peptide-2 (GLP-2) analogue therapy have highlighted the importance of remnant intestinal location, including the colon [22]. GLP-2 is mainly secreted from the ileum and colon [23], while its receptors are highly expressed in the jejunum [24]. Therefore, the length and the segmental location of the remnant bowel may affect adaptation and therapeutic efficacy. Our model, which preserves the ileocecal valve while resecting the distal ileum, provides a useful platform for investigating such segment-specific roles in SBS.

This study had some limitations. First, the absorption of nutrients in the colon was not quantified. However, there was a significant increase in *Asbt* expression in the colon, whereas there was no difference in the BA concentration in the feces, suggesting that possible absorption occurs in the colon to some extent. These findings reiterate the importance of the colon in patients with SBS. Second, it should be noted that the results of this study were based on a single time point at 14 days postoperatively. Longer term studies may reveal further functional changes in the colon. Third, it is currently unclear whether changes in the expression of BA absorption-related genes in the colon are due to resection of the ileum or secondary to changes in the gut microbial community. BAs are metabolic and immune signaling molecules synthesized from cholesterol in the liver and are transported to the intestine, where they are metabolized by gut bacteria. Therefore, the relationship between BA metabolism and the gut microbiota is critical. Additionally, the impact of IR on BA enterohepatic circulation and its subsequent effects on the liver may vary depending on the presence of the cecum [25, 26]. Considering that the cecum is a large reservoir of bacteria within the gut, preserving the cecum in IR models could be crucial for the further advancement of these studies. Therefore, future studies should aim at elucidating the changes in the gut microbiota and the differences in functional adaptation within the colon following ICR and IR, with a focus on the postoperative period.

## 5. Conclusions

This study involving IR with preservation of the cecum in mice revealed significant morphological and functional changes in the colon, especially in the proximal colon. These findings suggest that the colon may play a crucial role in adaptation following IR, highlighting its potential significance in a compensatory mechanism following massive small bowel resection, considering BA absorption.

## Figures and Tables

**Table 1:** Primers used for real-time polymerase chain reaction analysis of messenger RNA expression.

**Gene name**	**Forward**	**Reverse**	
*Asbt*	TTGCCTCTTCGTCTACACC	CCAAAGGAAACAGGAATAACAAG	IDT
*Fabp6*	ACGTGATTGAAAGGGGACGTAACTT	CATTCTTTGCCAATGGTGAACTTGT	Thermo Fisher
*Fxr*	CTCTGCTCACAGCGATCGTC	CACCGCCTCTCTGTCCTTGA	Operon
*Ostb*	GTATTTTCGTGCAGAAGATGCG	TTTCTGTTTGCCAGGATGCTC	IDT

## Data Availability

All data generated or analyzed during this study are included in this article. Further inquiries can be directed to the corresponding author.
